# Transcriptome data and gene ontology analysis in human macrophages ingesting modified lipoproteins in the presence or absence of complement protein C1q

**DOI:** 10.1016/j.dib.2016.09.008

**Published:** 2016-09-14

**Authors:** Minh-Minh Ho, Deborah A. Fraser

**Affiliations:** Department of Biological Sciences, California State University, Long Beach, CA 90840, USA

**Keywords:** Complement, Atherosclerosis, Lipoprotein, Macrophage

## Abstract

We characterized the transcriptional effects of complement opsonization on foam cell formation in human monocyte-derived macrophages (HMDM). RNA-sequencing was used to identify the pathways modulated by complement protein C1q during HMDM ingestion of the atherogenic lipoproteins oxidized low density lipoprotein (oxLDL) and acetylated low density lipoprotein (acLDL). All raw data were submitted to the MIAME-compliant database Gene Expression Omnibus (accession number GEO: GSE80442; http://www.ncbi.nlm.nih.gov/geo/query/acc.cgi?acc=GSE80442). Data presented here include Venn diagram overviews of up- and down-regulated genes for each condition tested, gene ontology analyses of biological processes, molecular functions and cellular components and KEGG pathway analysis. Further investigation of the pathways modulated by C1q in HMDM during ingestion of atherogenic lipoproteins and their functional relevance are described in “Macrophage molecular signaling and inflammatory responses during ingestion of atherogenic lipoproteins are modulated by complement protein C1q” (M.M. Ho, A. Manughian-Peter, W.R. Spivia, A. Taylor, D.A. Fraser, 2016) [1].

**Specifications Table**

**Table:** 

Subject area	*Biology*
More specific subject area	*Complement and Atherosclerosis*
Type of data	*Tables, Figure*
How data was acquired	*RNA-sequencing was performed using Illumina HiSeq 2500. Gene expression data were input into the DAVID online tool for Gene Ontology and KEGG Pathway analysis.*
Data format	*Analyzed, raw*
Experimental factors	*RNA was isolated from human monocyte-derived macrophages (HMDM) incubated with either oxidized (oxLDL) or acetylated low-density lipoprotein (acLDL) in the presence or absence of C1q.*
Experimental features	*RNA-seq analysis was performed and data subjected to gene ontology analysis to identify biological processes, molecular functions and cellular components modulated by C1q*
Data source location	*Long Beach, CA*
Data accessibility	*Analyzed data is within this article and raw data is available at the NCBI database at GEO series accession number GEO:*GSE80442*,*http://www.ncbi.nlm.nih.gov/geo/query/acc.cgi?acc=GSE80442

**Value of the data**

• These data provide a list of all genes modulated in human macrophages during foam cell formation.
• These data may be used to identify the effect of complement C1q opsonization on macrophage gene expression.
• Gene ontology analysis identifies pathways that may provide therapeutic targets for restoring defective foam cell removal in atherosclerosis.

## 1 Data

**Fig. 1:**
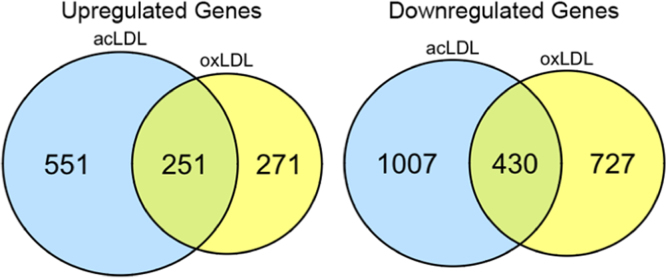
Overlap of genes modulated by C1q. HMDM pooled from 10 healthy donors were incubated with 10 µg protein/ml oxLDL or acLDL in the absence or presence of 75 µg/ml C1q for 3 h at 37 °C in triplicate. Differentially expressed genes from RNA-sequencing were determined using Cyber-T software (*n*=3, *p*<0.05, *t*-test). Libraries were compared to each other to show the intersection of all significant genes upregulated, or downregulated by C1q between acLDL or oxLDL treatment.

The data shown here include quantification of genes that were up or down-regulated by complement protein C1q in macrophages during ingestion of the atherogenic lipoproteins oxLDL and acLDL and gene ontology analysis. Overlapping upregulated and downregulated genes in the presence of C1q are visualized in Venn diagrams (Fig. 1). Data presented include gene ontology analysis based on biological processes of all significantly modulated genes (Table 1), upregulated genes (Table 2), and downregulated genes (Table 3) due to C1q during ingestion of oxLDL or acLDL and the overlap of the genes in common between lipoprotein treatment. Gene ontology analysis of all modulated genes based on molecular function (Table 4) and cellular component (Table 5) are also provided. Table 6 includes KEGG pathway analysis of all C1q modulated genes based on canonical pathways.

## 2 Experimental design, materials and methods

### 2.1 Experimental design

To examine and identify biological processes modulated by C1q during ingestion of modified lipoproteins in an unbiased manner, mRNA was collected from human monocyte-derived macrophages treated with physiologically relevant concentrations of oxidized and acetylated forms of LDL alone, or opsonized with C1q. RNA-seq was performed to identify genes that were up- or down-regulated by C1q in macrophages during ingestion of these atherogenic modified lipoproteins.

### 2.2 Cell isolation and lipoprotein treatment

Human monocyte-derived macrophages (HMDM) were prepared from human blood of 10 donors, according to the guidelines and approval of California University Long Beach (CSULB) Institutional Review Board and as described [2], [3]. RNA was isolated from untreated HMDM or HMDM treated with 10 µg protein/ml oxLDL or acLDL alone, or opsonized with 75 µg/ml C1q. Cells were incubated at 37 °C for 3 h in 5% CO_2_ as described [1].

### 2.3 RNA isolation and RNA-seq

RNA was isolated and RNA-seq was performed as described [1].

### 2.4 Data analysis

Statistically significant differences in gene expression (*p*<0.05) were determined using UCI׳s CyberT in-house software [4]. The overlap of genes determined to be up- or down-regulated by C1q during acLDL or oxLDL treatment was shown with Venn diagrams (Fig. 1). Gene lists of all significantly modulated genes by C1q during ingestion of oxLDL or acLDL (*p*<0.05) were used as input for gene ontology (GO) analysis (Table 2, Table 4, Table 5) or KEGG pathway analysis (Table 6) using DAVID (https://david.ncifcrf.gov/) [5]. In addition, resulting upregulated (Table 2) and downregulated (Table 3) gene lists were also used as input separately in DAVID. An adjusted EASE (Expression Analysis Systemic Explore Score) score of 0.05 and a threshold count of >2 genes were used. Benjamini–Hochberg multiple testing correction was applied to the *p-*values. GO terms with FDR *q*<0.05 were considered significantly enriched within the gene set. The overlap between oxLDL and acLDL gene sets for each GO term was also determined.

## Figures and Tables

**Table 1:** Gene ontology analysis of all C1q modulated genes based on biological processes.

**Biological Processes GO Term: All Genes**	**Number of genes in the gene set**		
	***p*****<0.05**		**Overlap oxLDL/acLDL**
	**oxLDL**	**acLDL**	
Immune response	105⁎	98	39
Defense response	87⁎	88	38
Inflammatory response	56⁎	55	23
Response to wounding	66⁎	73	28
Positive regulation of immune system process	37⁎	37	15
Positive regulation of cell activation	22⁎	26	10
Regulation of transcription	–	324⁎	–
Anti-apoptosis	–	44⁎	–
RNA processing	53	88⁎	26
Programmed cell death	–	95⁎	–
Cell death	62	108⁎	37
Death	62	108⁎	37
Apoptosis	–	93⁎	–
Transcription	–	261⁎	–
tRNA metabolic process	17	28⁎	7
ncRNA metabolic process	28	44⁎	12
I-kappaB kinase/NF-kappaB cascade	10	19⁎	7
Positive regulation of protein kinase cascade	27	35⁎	12
Regulation of I-kappaB kinase/NF-kappaB cascade	19	26⁎	9
Regulation of programmed cell death	71	115⁎	32

⁎ FDR *q*<0.05.

**Table 2:** Gene ontology analysis of all C1q upregulated genes based on biological processes.

**Biological processes GO term upregulated genes**	**Number of genes in the gene set**		
	***p*****<0.05**		**Overlap oxLDL/acLDL**
	**oxLDL**	**acLDL**	
Anti-apoptosis	–	27⁎	–
Positive regulation of cellular biosynthetic process	31	54⁎	21
Regulation of transcription from RNA polymerase II promoter	27	56⁎	18
Positive regulation of biosynthetic process	31	54⁎	21
Intracellular signaling cascade	52⁎	81⁎	31
Positive regulation of nitrogen compound metabolic process	28	50⁎	19
Positive regulation of macromolecule biosynthetic process	28	50⁎	20
Apoptosis	–	47⁎	–
Programmed cell death	–	47⁎	–
Cell death	–	52⁎	–
Protein kinase cascade	18	33⁎	13
Regulation of transcription	–	138⁎	–
Positive regulation of transcription, DNA-dependent	22	39⁎	15
Death	–	52⁎	–
Positive regulation of RNA metabolic process	22	39⁎	15
Regulation of programmed cell death	–	56⁎	–
Positive regulation of NF-kappaB transcription factor activity	–	10⁎	–
Regulation of cell death	–	56⁎	–
I-kappaB kinase/NF-kappaB cascade	–	12⁎	–
Regulation of apoptosis	–	55⁎	–

⁎ FDR *q*<0.05.

**Table 3:** Gene ontology analysis of all C1q downregulated genes based on biological processes.

**Biological Processes GO Term Downregulated Genes**	**Number of genes in the gene set**		
	***p*****<0.05**		**Overlap oxLDL/acLDL**
	**oxLDL**	**acLDL**	
Immune response	79⁎	–	–
Defense response	59⁎	–	–
Inflammatory response	37⁎	–	–
Response to virus	17⁎	15	–
ncRNA metabolic process	24	40⁎	12
tRNA metabolic process	14	26⁎	7
RNA processing	39	67⁎	19
DNA repair	–	42⁎	–
ncRNA processing	19	30⁎	10
Response to DNA damage stimulus	–	47⁎	–
DNA metabolic process	–	57⁎	–
Translation	26	41⁎	12

⁎ FDR *q*<0.05.

**Table 4:** Gene ontology analysis of all C1q modulated genes based on molecular function.

**Molecular function GO term all genes**	**Number of genes in the gene set**		
	***p*****<0.05**		**Overlap oxLDL/acLDL**
	**oxLDL**	**acLDL**	
RNA binding	72	110⁎	41
Zinc ion binding	–	285⁎	
Transition metal ion binding	–	329⁎	
DNA binding	–	281⁎	

⁎ FDR *q*<0.05.

**Table 5:** Gene ontology analysis of all C1q modulated genes based on cellular component.

**Cellular component GO term: All genes**	**Number of genes in the gene set**		
	***p*****<0.05**		**Overlap oxLDL/acLDL**
	**oxLDL**	**acLDL**	
Intracellular organelle lumen	145	255⁎	75
Membrane-enclosed lumen	149	262⁎	77
Organelle lumen	146	256⁎	75
Nuclear lumen	123	214⁎	68
Nucleolus	69	116⁎	36
Intracellular non-membrane-bounded organelle	–	304⁎	
Non-membrane-bounded organelle	–	304⁎	
Ribonucleoprotein complex	–	84⁎	
Nucleoplasm	–	123⁎	
Cytosol	111	166⁎	49
Miitochondrion	82	133⁎	38

⁎ FDR *q*<0.05.

**Table 6:** KEGG analysis of all C1q modulated genes based on canonical pathways.

**KEGG Canonical Pathways: All Genes**	**Number of genes in the gene set**		
	***p*****<0.05**		**Overlap oxLDL/acLDL**
	**oxLDL**	**acLDL**	
Toll-like receptor signaling pathway	16	23	7
Apoptosis	14	20	8
RIG-I-like receptor signaling pathway	–	17	–
Ubiquitin mediated proteolysis	–	26	–
Pyrimidine metabolism	–	19	–
NOD-like receptor signaling pathway	11	14	6
Acute myeloid leukemia	–	13	–
Neurotrophin signaling pathway	–	21	–
B cell receptor signaling pathway	–	14	–
Arginine and proline metabolism	–	11	–
Small cell lung cancer	–	15	–
Aminoacyl-tRNA biosynthesis	–	9	–
Cytokine-cytokine receptor interaction	38	–	–
Systemic lupus erythematosus	17	–	–
Jak-STAT signaling pathway	21	–	–
RIG-I-like receptor signaling pathway	12	–	–
Chemokine signaling pathway	23	–	–
